# 

**Published:** 1878-12

**Authors:** 


*CONTENTS.*

| | Page | | Page |
|---|---|---|---|
| Our Quarter Centennial | 659 | A Patent and Patient Mother | 676 |
| Constipation | 660 | To Cure Colds | 676 |
| Moles, Warts, Etc. | 661 | Dieting for Health | 677 |
| Discernment | 662 | Bad Temper and Insanity | 678 |
| Indecision | 662 | Hardening the Constitution | 679 |
| Heart Disease | 663 | N. Y. and London Mortality | 679 |
| Weak Eyes | 663 | Peter's Wife's Mother | 680 |
| Drunken Women | 664 | Winter Shoes | 681 |
| Metamorphoses | 664 | When Began We | 681 |
| Catarrh | 668 | Don't Sleep Well | 684 |
| Moral Hygiene | 670 | Restless Nights | 685 |
| True Politeness | 671 | Lives of Medical Men | 686 |
| Cheerfulness | 671 | Dangerous Water Pipes | 687 |
| Jesting | 672 | | |

